# Supplementary material for: The Oncoprotein Fra-2 Drives the Activation of Human Endogenous Retrovirus Env Expression in Adult T-Cell Leukemia/Lymphoma (ATLL) Patients
Source: Cells. 2024 Sep 10;13(18):1517. doi: 10.3390/cells13181517 (PMC11430398; doi:10.3390/cells13181517)
Supplement: Supplementary file 1 [file cells-13-01517-s001.zip › Tram et al. Appendix A- Supplementary Figures.pdf]

Appendix A-SUPPLEMENTARY FIGURES LEGENDS

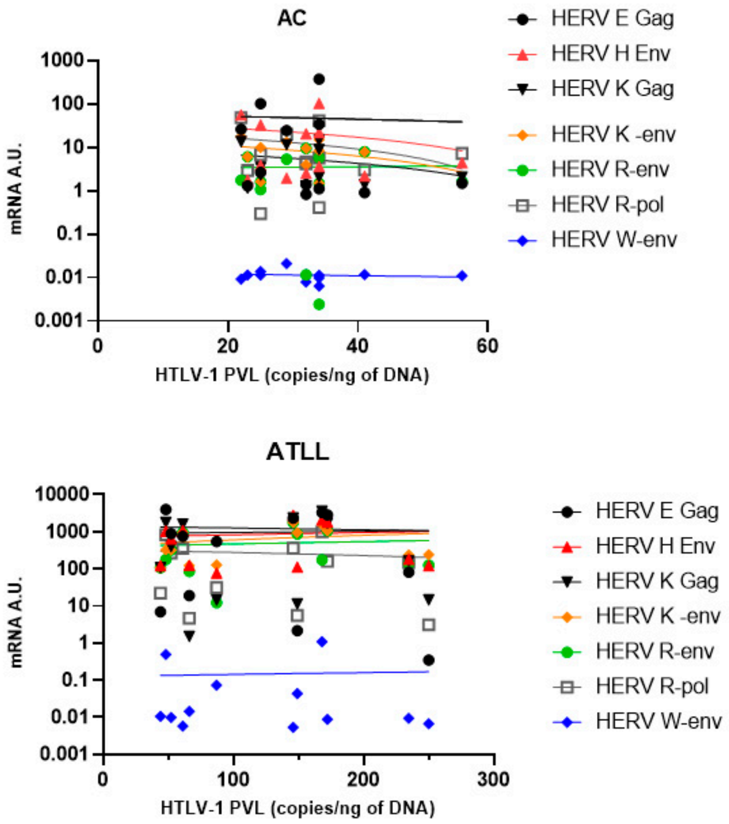

Supplemental Figure 1 related to Figure 3

Supplemental Figure 1: The PVL load in ATL cells does not correlate with the expression of HERV genes. The Relevance of the PVL of HTLV-1 and HERV env mRNA expression in asymptomatic carriers (AC) (A) and ATL patients (B) was analyzed using a Pearson correlation Test.

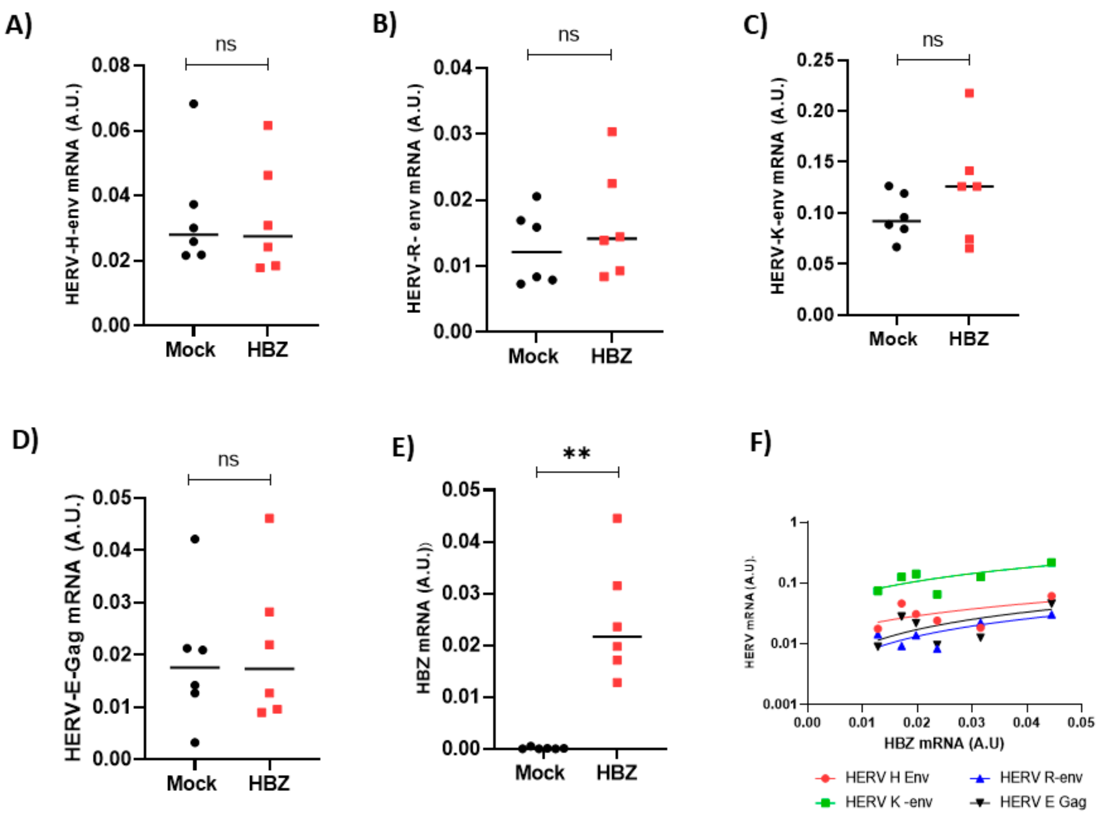

Supplemental Figure 2 related to Figure 4

Supplemental Figure 2: HERVs env mRNA expression is not increased in HEK293 cells stably expressing HBZ. (A-E) HEK293 cells stably expressing HBZ were harvested at different passages (from p3 to p8), and expression of HBZ and HERVs mRNA was assessed by qRT-PCR. l. (F) The Relevance of the HBZ and HERV env mRNA expression in 293 cells stably expressing HBZ was analyzed using a Pearson correlation Test.

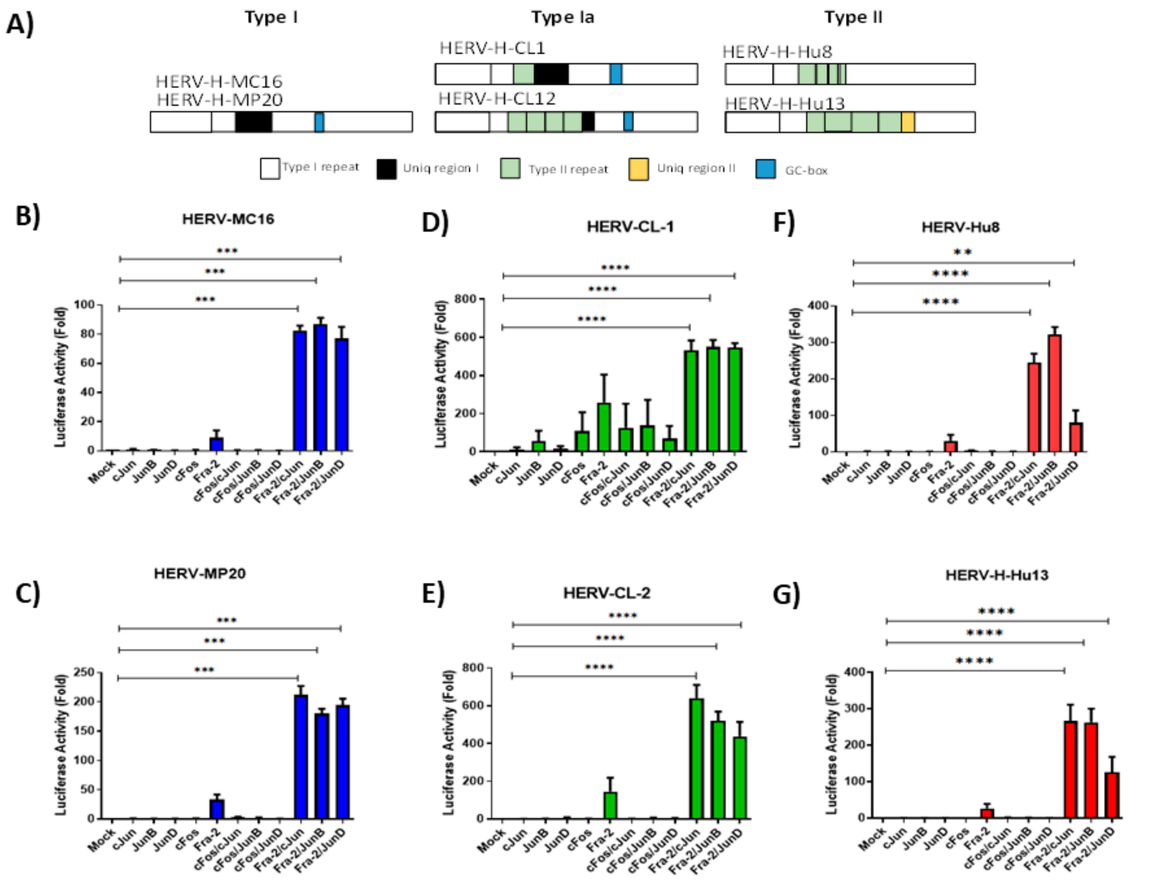

Supplemental Figure 3 realted to Figure 5

Supplemental Figure 3: Fra-2 but not cFos activate type I and II HERV-H LTR of (A) Schematic representation of the different types of HERV-H LTR. (B-G) HEK293T cells were co-transfected with a plasmid carrying the luciferase reporter gene under the control of the different HERV-H 5′LTRs, AP-1 expression vectors, and pRcActin-LacZ. At 48 hr. post-transfection, cells were harvested and assayed for luciferase activity. Results are shown as a fold increase of the Mock control and represent the mean values of three independently transfected cell samples (one-way ANOVA test with Dunn's multiple comparisons post-test * $p \leq 0.01$; ** $p \leq 0.001$).

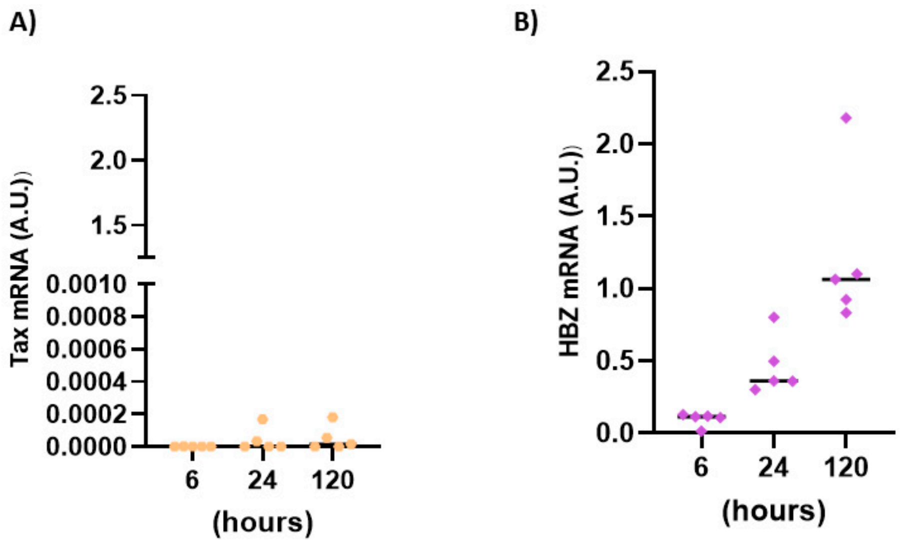

Supplemental Figure 4 related to Figure 8

Supplemental Figure 4: Kinetic analysis of Tax and HBZ env mRNA in CD8+-depleted PBMCs from asymptomatic carriers and ATL patients. CD8+-depleted PBMCs from 5 ATL patients were cultivated *ex vivo* for five days. Tax (A) and HBZ (B) mRNA were quantified at different time points using qRT-PCR.

Supplementary Table S1: List of primers for qRT-PCR

Primer name	Primer sequence (5‘-3‘)
**HERV-K gag-Fwd**	GGCCATCAGAGTCTAAACCACG
**HERV-K gag-Rev**	GCAGCCCTATTTCTTCGGACC
**HERV-R pol Fwd**	GGGCCAATTATGCTTACCAA
**HERV-R pol- Rev**	ATGGGCTGATCTGGCTCTAA
**HERV-R env Fwd**	CATGGGAAGCAAGGGAACT
**HERV-R env Rev**	CTTTCCCCAGCGAGCAATAC
**HERV-K env Fwd**	CGACTTAACAGAAAGTTTAGACAAAC
**HERV-K env Rev**	GCTGTGACTGCAATTAATCCC
**HERV-E gag Fwd**	CACATGGTGGAGAGTCGTGTTT
**HERV-E gag Rev**	GCTTGCGGCTTTTCAGTATAGG
**HERV-W env Fwd**	TTCACTGCCCACACCCAT
**HERV-W env Rev**	GAGGTACCACAGACAAAAAATATTCCT
**HERV-H env Fwd**	AGGGCACCCTCCAATACTTC
**HERV-H env Rev**	AGAAGCGGCTAGGAGAGAATG
**HTLV-1-Tax Fwd**	CCAACACCATGGCCCACTT
**HTLV-1-Tax Rev**	GATGGGGTCCCAGGTGATCT
**HTLV-1-HBZ Fwd**	TGGCGGCCTCAGGGCTGT-
**HTLV-1-HBZ Rev**	GGAGGGCCCCGTCGCAG
**Fra-2 Fwd**	CACTCCGGGCACCTCGAACC
**Fra-2 Rev**	CCAGCAGAGTGGGGGAGTTC
**HPRT-1 Fwd**	GACACTGGCAAAACAATGCA
**HPRT-1 Rev**	GGTCCTTTTCACCAGCAAGCT-

Supplementary Table S2: List of primers for ChIP qRT-PCR

Primer name	Primer sequence (5‘-3‘)
**HERV-H-HU13 (type II) Fwd**	GGGACCTGCACGTATACATC
**HERV-H-HU13 (type II) Rev:**	CCTTCTTAAGGGTGGGAGAG
**HERV-H MC16 (Type I) Fwd**	GGTTCCTGCCTTAACTGATG
**HERV-H MC16 (Type I) Rev:**	AGAGTCAGGGAAGGGAGATG
**HERV-H-L19 Fwd**	GCGACCTGCACATATACATC
**HERV-H-L19 Rev**	GCCAGGAGAAGGAATTTCAC
**HERV-H-MP20 Fwd**	CTGCACGTACACATCCAG 3'
**HERV-H-MP20 Rev:**	CCAGGAGGAGGAATTTCAC
**b-Globin Fwd**	AGGCTGCTGGTTGTCTACCCTTG
**b-Globin Rev**	AGCTCACTGAGGCTGGCAAAGGTG
