# Supplementary material for: The Oncoprotein Fra-2 Drives the Activation of Human Endogenous Retrovirus Env Expression in Adult T-Cell Leukemia/Lymphoma (ATLL) Patients
Source: Cells. 2024 Sep 10;13(18):1517. doi: 10.3390/cells13181517 (PMC11430398; doi:10.3390/cells13181517)
Supplement: Supplementary file 1 [file cells-13-01517-s001.zip › Tram et al. Appendix B- Supplementary Tables.pdf]

Supplementary Table S1: List of primers for qRT-PCR

Primer name	Primer sequence (5'-3')
**HERV-K gag-Fwd**	GGCCATCAGAGTCTAAACCACG
**HERV-K gag-Rev**	GCAGCCCTATTTCTTCGGACC
**HERV-R pol Fwd**	GGGCCAATTATGCTTACCAA
**HERV-R pol- Rev**	ATGGGCTGATCTGGCTCTAA
**HERV-R env Fwd**	CATGGGAAGCAAGGGAACT
**HERV-R env Rev**	CTTTCCCCAGCGAGCAATAC
**HERV-K env Fwd**	CGACTTAACAGAAAGTTTAGACAAAC
**HERV-K env Rev**	GCTGTGACTGCAATTAATCCC
**HERV-E gag Fwd**	CACATGGTGGAGAGTCGTGTTT
**HERV-E gag Rev**	GCTTGCGGCTTTTCAGTATAGG
**HERV-W env Fwd**	TTCACTGCCCACACCCAT
**HERV-W env Rev**	GAGGTACCACAGACAAAAAATATTCCT
**HERV-H env Fwd**	AGGGCACCCTCCAATACTTC
**HERV-H env Rev**	AGAAGCGGCTAGGAGAGAATG
**HTLV-1-Tax Fwd**	CCAACACCATGGCCCACTT
**HTLV-1-Tax Rev**	GATGGGGTCCCAGGTGATCT
**HTLV-1-HBZ Fwd**	TGGCGGCCTCAGGGCTGT-
**HTLV-1-HBZ Rev**	GGAGGGCCCCGTCGCAG
**Fra-2 Fwd**	CACTCCGGGCACCTCGAACC
**Fra-2 Rev**	CCAGCAGAGTGGGGGAGTTC
**HPRT-1 Fwd**	GACACTGGCAAAACAATGCA
**HPRT-1 Rev**	GGTCCTTTTCACCAGCAAGCT-

Supplementary Table S2: List of primers for ChiP qRT-PCR

Primer name	Primer sequence (5'-3')
**HERV-H-HU13 (type II) Fwd**	GGGACCTGCACGTATACATC
**HERV-H-HU13 (type II) Rev:**	CCTTCTTAAGGGTGGGAGAG
**HERV-H MC16 (Type I) Fwd**	GGTTCCTGCCTTAACTGATG
**HERV-H MC16 (Type I) Rev:**	AGAGTCAGGGAAGGGAGATG
**HERV-H-L19 Fwd**	GCGACCTGCACATATACATC
**HERV-H-L19 Rev**	GCCAGGAGAAGGAATTTCAC
**HERV-H-MP20 Fwd**	CTGCACGTACACATCCAG 3'
**HERV-H-MP20 Rev:**	CCAGGAGGAGGAATTTCAC
**b-Globin Fwd**	AGGCTGCTGGTTGTCTACCCTTG
**b-Globin Rev**	AGCTCACTGAGGCTGGCAAAGGTG
